# Case Report: Clinical characteristics and management strategies of intraspinal tophaceous gout: a case series and literature review

**DOI:** 10.3389/fmed.2026.1961092

**Published:** 2026-10-07

**Authors:** Hongyan Zhang, Liping Lu, Jianguo Tian, Zhenzhen Ma

**Affiliations:** 1 Department of Pathology, Shengli Oilfield Central Hospital, Dongying, Shandong, China; 2 Department of Pathology, Peking University People’s Hospital, Beijing, China; 3 Department of Medical Imaging, Shengli Oilfield Central Hospital, Dongying, Shandong, China; 4 Department of Rheumatology and Immunology, Shengli Oilfield Central Hospital, Dongying, Shandong, China

**Keywords:** dual-energy computed tomography, monosodium urate, postoperative hematoma, spinal decompression, spinal epidural mass, tophaceous gout, urate-lowering therapy

## Abstract

**Background:**

Intraspinal tophaceous gout, defined as monosodium urate crystal deposition within the effective spinal canal causing neural compression, is a rare subtype of spinal gout with no unified diagnostic standard. Despite documented gout history, it is frequently misdiagnosed preoperatively.

**Methods:**

We retrospectively analyzed five male patients with pathologically confirmed intraspinal tophaceous gout (median age 25 years, range 21–48) admitted to two tertiary hospitals between January 2018 and May 2026, alongside a systematic literature review of strictly defined cases.

**Results:**

The preoperative misdiagnosis rate was 60%. Computed tomography values of 142–272 Hounsfield units provided diagnostic clues, yet no patient underwent preoperative dual-energy computed tomography. Three patients underwent open decompression with fusion, one endoscopic decompression, and one computed tomography-guided biopsy followed by conservative observation; pathology confirmed gouty tophus or granuloma in all. One patient developed a postoperative intraspinal hematoma on day 4, a previously unreported complication. Long-term follow-up revealed multiple vertebral compression fractures and thoracic spinal cord injury in one patient with poor compliance with urate-lowering therapy, a bone event that requires long-term vigilance. Pooled analysis of the 38 strictly defined cases (33 literature cases plus this series) confirmed that the preoperative misdiagnosis rate exceeds 50% and that most lesions are epidural.

**Conclusion:**

Intraspinal tophaceous gout should be considered in young male patients with back pain and neural deficits, even with known gout history. A stepwise imaging strategy, individualized surgical or biopsy-first management, vigilant postoperative monitoring, and long-term urate-lowering therapy are essential for optimal outcomes.

## 1 Introduction

Gout is a crystal arthropathy caused by the deposition of monosodium urate (MSU) crystals. In addition to peripheral joints and soft tissues, it can also affect the axial skeleton, a condition known as spinal gout or axial gout (1, 2). Since Kersley et al. reported the first case of spinal gout in 1,950, more than 300 cases have been documented worldwide. Patients are predominantly middle-aged and elderly men, with the lumbar spine being the most commonly affected segment (1, 2). However, among the various subtypes of spinal gout, tophaceous gout directly deposited within the spinal canal (intraspinal tophaceous gout) causing compression of the spinal cord, nerve roots, or cauda equina is exceedingly rare (3, 4).

Currently, there is no internationally unified diagnostic standard or anatomical classification for intraspinal tophaceous gout (5). In this study, we adopt a strict definition: MSU crystal deposition located within the effective volume of the spinal canal (including the epidural space, subdural space, intramedullary region, and intradiscal lesions protruding into the canal), presenting as a space-occupying lesion with spinal canal compression and neural compression as the primary clinical manifestations. The rationale for this strict definition is that intraspinal lesions differ fundamentally from vertebral/appendage lesions in terms of management strategy, misdiagnosis spectrum, and prognostic assessment criteria.

Existing literature on intraspinal tophaceous gout consists almost exclusively of single case reports, and the reported patients are generally older. This study presents 5 patients with strictly defined intraspinal tophaceous gout from two tertiary hospitals, representing one of the larger case series to date. Notably, our cohort is characterized by a significantly younger age (median 25 years). Additionally, we report a postoperative intraspinal hematoma, a complication not previously described in the literature. Combined with 33 literature cases, this study aims to: (1) analyze preoperative diagnostic pitfalls; (2) compare minimally invasive and open surgical strategies; (3) report the occurrence and management of postoperative intraspinal hematoma; and (4) emphasize the importance of postoperative rheumatology-led urate-lowering therapy and DECT assessment.

## 2 Materials and methods

### 2.1 Inclusion criteria

This retrospective case series included patients with intraspinal tophaceous gout admitted to two tertiary hospitals (Shengli Oilfield Central Hospital and Peking University People's Hospital) between January 2018 and May 2026. The inclusion criteria were: (1) postoperative histopathological confirmation of gouty tophus or gouty granuloma; (2) preoperative imaging (CT/MRI) and surgical records clearly indicating the lesion located within the effective spinal canal volume (epidural, subdural, intramedullary, or intradiscal protrusion into the canal), with compression of the dural sac, spinal cord, or nerve roots; and (3) clinical manifestations of nerve root or spinal cord compression symptoms (e.g., limb numbness, weakness, radiating pain, intermittent claudication, or spinal cord injury signs). Exclusion criteria were: (1) lesions involving only the vertebral body, articular processes, lamina, transverse processes, spinous processes, or ligaments without intraspinal extension; (2) absence of spinal canal occupation and neural compression evidence; and (3) incomplete clinical or pathological data.

The same inclusion and exclusion criteria were applied not only to the center cases but also, wherever the relevant information was available in the original publications, to the literature cases included in the systematic review.

### 2.2 Data collection and follow-up

Demographic data, gout history, initial symptoms, affected segments, preoperative diagnosis, imaging characteristics (CT values, MRI signals, DECT), surgical approach, pathological results, serum uric acid levels, comorbidities, postoperative complications, and follow-up data were collected. Follow-up methods included outpatient review, telephone follow-up, and imaging re-examination. The last follow-up was in May 2026.

### 2.3 Literature search strategy

A systematic search of PubMed and Embase was performed up to May 2026. The English search terms were: “intraspinal gout”, “intraspinal tophus”, “spinal tophaceous gout”, and “spinal canal gout”. Two researchers independently screened the literature and cross-checked the results. Case reports or case series explicitly reporting intraspinal tophaceous gout (not merely vertebral/paravertebral involvement) were included. Reviews, conference abstracts, and incomplete data were excluded. To ensure a consistent definition of “intraspinal” across different publications, two reviewers independently assessed each retrieved case against the same strict anatomical criteria applied to the center cases (lesion within the effective spinal canal volume with neural compression), based on the original description of lesion location in the text and figures; cases in which the intraspinal location could not be unambiguously confirmed were excluded. Disagreements were resolved by discussion and, when necessary, by consultation with a third senior investigator. Clinical data were extracted independently by the two reviewers using a standardized form and cross-checked for accuracy. Ultimately, 33 literature cases meeting the strict definition (lesion within the effective spinal canal volume with neural compression) were included (Supplementary Table 1).

## 3 Results

### 3.1 Clinical characteristics

All 5 patients were male, aged 21–48 years (median 25 years), and all had a history of gout (duration 1–20 years). The initial symptoms were all back pain with neural compression manifestations: 2 cases with bilateral lower limb numbness/weakness, and 3 cases with unilateral lower limb radiating pain/numbness. Affected segments: thoracic spine in 1 case (T10/11), thoracolumbar involvement in 1 case (T11–12 + L5), and lumbar spine in 3 cases (L4–S1, L4–L5, L3/4–L5/S1). Preoperative misdiagnosis occurred in 3 cases (60%): thoracic disc herniation/spinal stenosis (Patient 1), lumbar disc herniation/spinal stenosis (Patient 4), and malignant tumor/metastasis (Patient 5).

### 3.2 Imaging characteristics

Preoperative CT showed slightly hyperdense or mixed-density lesions. MRI mostly demonstrated hypointense or isointense signals on T1-weighted images and hypointense or mixed signals on T2-weighted images, overlapping with the signals of epidural tumors or disc herniation. In Patient 1, preoperative thoracic CT showed T10/11 disc herniation and posterior vertebral osteophyte formation with spinal stenosis at the same level, without typical hyperdense tophus. In Patient 2, preoperative CT revealed a hyperdense intraspinal lesion at L4–S1 with CT values of approximately 142–272 HU, leading to a preoperative suspicion of tophaceous gout (detailed imaging findings are presented in the case description below and in Figures 1D–G). In Patient 3, preoperative CT showed multiple appendage destruction at T11–T12 with extension into the epidural space and compression of the dural sac, as well as a mass in the spinal canal (epidural) at L5. In Patient 4, preoperative CT and MRI showed L4–L5 spinal stenosis and disc herniation, with an abnormal-density lesion in the epidural space. In Patient 5, preoperative MRI showed strip-like long T1 and long T2 signals around the L3/4–L5/S1 facet joints, bone destruction, and protrusion of the lesion into the spinal canal.

**Figure 1:**
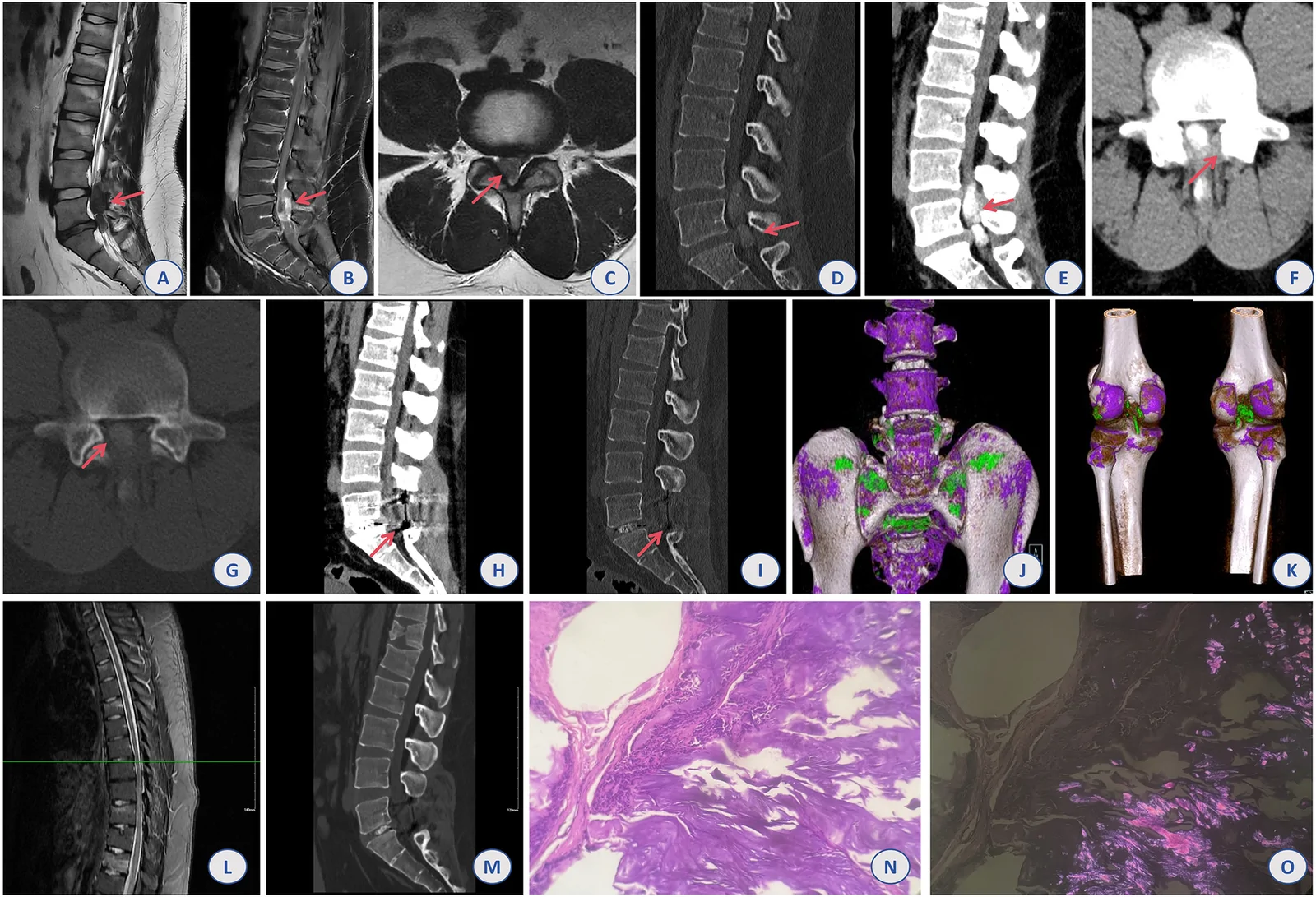
Patient 2: imaging and pathological findings. **(A,B)** Preoperative MRI (sagittal T1WI and T2WI): Mass in the spinal canal at L5–S1, well-defined, hypointense on T2WI, spinal stenosis at the same level, closely related to the dural sac with cauda equina compression (arrows). **(C)** Preoperative contrast-enhanced MRI (axial): Obvious eccentric enhancement at the upper margin of the lesion, mild enhancement in the remaining portions, with better delineation of boundaries than on non-contrast images (arrow). **(D–G)** Preoperative CT (sagittal and axial bone windows): Two oval-shaped hyperdense lesions in the spinal canal at L5–S1, well-defined, CT values approximately 142–272 HU, spinal stenosis and nerve compression at the same level (arrows). **(H,I)** Postoperative CT (sagittal): Postoperative changes at L5, spinal canal enlargement, bone defect, and a small amount of gas in the intervertebral space after L5–S1 implantation (arrows). **(J,K)** Dual-source DECT 3D reconstruction: Multiple irregular patchy and massive urate crystal deposits (green color-coded) around the sacroiliac joints, L5 vertebra, and both knees. **(L)** Thoracic MRI in January 2026 (sagittal T2WI): T3, T5, T10, and T12 vertebrae slightly flattened with heterogeneous signal, consistent with compression fractures; T6–T10 discs mildly posteriorly protruding with mild dural sac compression and abnormal thoracic spinal cord signal, suggestive of injury degeneration. **(M)** Lumbar CT in January 2026 (sagittal): Partial laminar defect at L5, postoperative changes after L5–S1 implantation, and T12 compression fracture. **(N)** Pathological HE staining (×100): Calcified degenerative fibrocartilage tissue surrounded by dense fibrous connective tissue and hyaline degeneration, with multinucleated giant cells, foreign body giant cells, and chronic inflammatory cell infiltration. **(O)** Polarized light microscopy (×100): Lobulated tophaceous deposits with strongly birefringent urate crystals.

Notably, none of the 5 patients underwent preoperative DECT. Patient 2 underwent whole-body DECT assessment 4 months postoperatively, revealing multiple tophaceous deposits in the lumbar spine, sacrum, bilateral iliac wings, and both knees, with a total volume of 33.17 cm^3^.

### 3.3 Surgical strategies and pathological confirmation

Three cases (Patient 2, 3, and 4) underwent open decompression, lesion resection, and fusion with internal fixation. One case (Patient1) underwent endoscopic decompression. One case (Patient 5) was highly suspected of malignancy preoperatively; CT-guided needle biopsy was performed first, and after pathological confirmation of gout, no further open surgery was undertaken. Postoperative pathology in all cases confirmed gouty tophus or gouty granuloma: microscopy revealed pink amorphous/crystalline material deposition surrounded by multinucleated giant cells, foreign body giant cells, and histiocytic reactions.

### 3.4 Postoperative complications and follow-up

Patient 1 developed an intraspinal hematoma on postoperative day 4 and underwent emergency endoscopic hematoma evacuation—a rare complication not previously reported in the literature. Patients 2–5 had no early postoperative complications. During long-term follow-up, Patient 2, who had a history of lumbar trauma and poor compliance with urate-lowering therapy, developed multiple vertebral compression fractures (T3, T5, T10, T12) and thoracic spinal cord injury degeneration at 20 months postoperatively. Against the background of trauma, long-term hyperuricemia may have exacerbated bone structural fragility; such bone events should be regarded as complications requiring long-term vigilance rather than as proven causal outcomes.

## 4 Case series

The demographic and clinical characteristics of all five patients are summarized in Table 1. Below we present detailed clinical courses of two cases (Patient 1 and 2) to illustrate the diagnostic challenges and therapeutic management of intraspinal tophaceous gout.

**Table 1:** Demographic and clinical characteristics of five patients with intraspinal tophaceous gout.

Characteristics	Patient 1	Patient 2	Patient 3	Patient 4	Patient 5
Age/Sex	23 years/M	21 years/M	25 years/M	48 years/M	32 years/M
Gout history	5 years	1 year	7 years	20 years	10 years
Initial symptom	Low back pain with bilateral lower limb numbness/weakness	Low back pain with right lower limb numbness	Low back pain with bilateral lower limb weakness	Low back pain with right lower limb radiating pain	Low back pain with left calf numbness
Affected level	T10/11	L4–S1	T11–12, L5	L4–L5	L3/4–L5/S1
Preoperative diagnosis	Thoracic spinal stenosis/disc herniation	Benign intraspinal tumor; tophaceous gout highly suspected on CT	Gouty spondylitis	Lumbar disc herniation/stenosis	Malignancy/metastasis
CT value (HU)	231	142–272	—	—	—
Surgical approach	Endoscopic decompression + nucleotomy; hematoma evacuation on POD 4	Open decompression + lesion resection + fusion	Open decompression + lesion resection + 3-level fusion	Open decompression + lesion resection + fusion	CT-guided biopsy only
Pathological diagnosis	Tophus (calcified nucleus pulposus with lobulated tophus deposition)	Gouty foreign-body granuloma	Gouty tophus	Gouty tophus	Gouty pathological change
Serum uric acid (*μ*mol/L)	727	836	433	504	634
Preoperative urate-lowering therapy	None (no standardized ULT)	None	None	None	None
Time to postoperative urate target	Not documented	Not documented	Not documented	Not documented	Not documented
DECT use and results	Not performed	Not performed preoperatively; postoperative DECT (4 months after surgery): multiple tophaceous deposits, total volume 33.17 cm^3^	Not performed	Not performed	Not performed
Comorbidities	None	Hypertension/hepatic impairment	Hypertension	Hypertension	Hypertension/nephrolithiasis
Postoperative complication	Intraspinal hematoma on POD 4	None; acute gout flare in both knees at 2 weak (crystal dissolution reaction)	None	None	None (no surgery); urate-lowering therapy by rheumatology
Follow-up/Outcome	1 month, significant recovery of motor/sensory function	20 months, multiple vertebral compression fractures and thoracic cord injury	2 months, no new neurological symptoms, no intraspinal tophus recurrence	1 month, complete symptom relief, no recurrence	3 months: partial relief, stable neurology, persistent mass; continued medical therapy, surgical candidacy under evaluation

### 4.1 Patient 1

A 23-year-old male was admitted to the spinal surgery department on April 10, 2024, with a 4-month history of back pain accompanied by bilateral lower limb numbness and weakness, worsening over the past month. He had a 5-year history of elevated uric acid and four episodes of acute joint swelling, but had not received standardized urate-lowering therapy preoperatively. Physical examination revealed mild thoracolumbar kyphosis, tenderness and percussion pain over the T10–L2 spinous processes and paraspinal regions. Bilateral lower limb skin sensation was decreased, more pronounced distally. Left lower limb muscle strength was grade IV, right lower limb grade III. Bilateral knee and Achilles tendon reflexes were hyperactive, and bilateral Babinski signs were positive (Frankel grade D). Laboratory tests showed serum uric acid of 727.0 μmol/L.

Imaging: MRI from an outside hospital (March 30, 2024) showed T10/11 and T11/12 disc herniation with spinal stenosis and thoracic spinal cord injury at the corresponding levels. Preoperative thoracic CT showed T10/11 disc herniation, posterior vertebral osteophyte formation, and spinal stenosis at the same level, without typical hyperdense tophus (Figures 2A,B).

**Figure 2:**
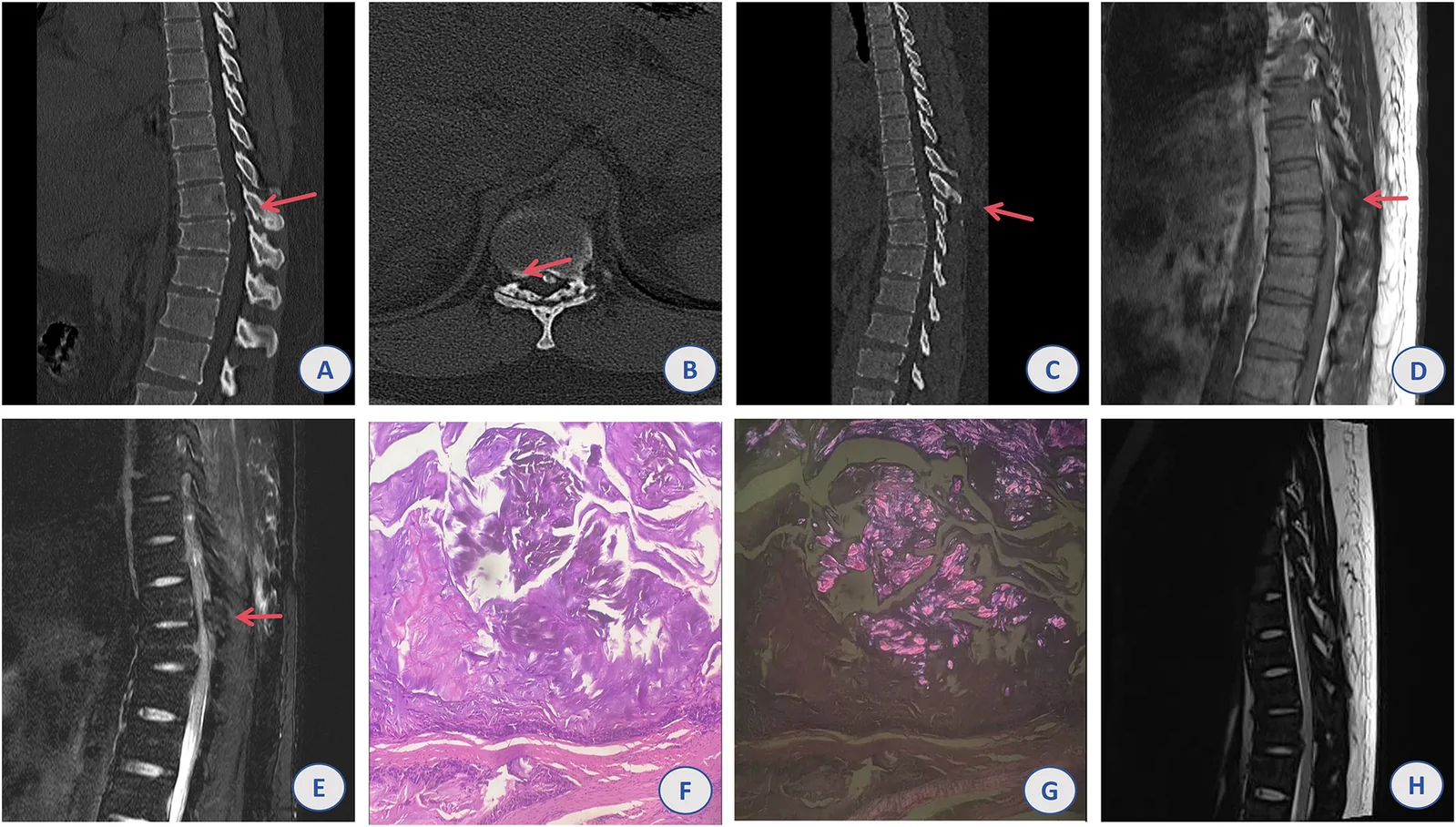
Patient 1: imaging and pathological findings. **(A,B)** Preoperative CT (sagittal and axial bone window): Oval-shaped free hyperdense lesion in the spinal canal at T10–11 (CT value approximately 231 HU), with smooth boundaries, spinal stenosis at the same level, and thoracic spinal cord compression (arrows). **(C)** Postoperative day 2 CT (sagittal): No residual hyperdense lesion in the spinal canal; no obvious thoracic bone abnormalities. **(D,E)** Postoperative day 4 MRI (sagittal T1WI and T2WI-FS): Partial laminar defect at T10; clumped hematoma on the left side of the spinal canal at T10–11 compressing the dural sac (arrows). **(F)** Pathological HE staining (×100): Extensively calcified degenerative fibrocartilage tissue surrounded by dense fibrous connective tissue and hyaline degeneration. **(G)** Polarized light microscopy (×100): Lobulated tophaceous deposits with strongly birefringent urate crystals. **(H)** Postoperative 1-month MRI (sagittal T2WI): Thoracic spinal cord compression relieved; no obvious abnormalities in the spinal canal or thoracic vertebrae.

Surgery: On April 13, 2024, under general anesthesia, posterior DELTA endoscopic T10/11 spinal canal decompression, protruding nucleus pulposus removal, and radiofrequency ablation were performed. On postoperative day 1, bilateral lower limb muscle strength recovered to grades IV–V. On postoperative day 2, CT re-examination showed no residual lesion in the spinal canal and no obvious bone abnormalities (Figure 2C). On postoperative day 3, the patient reported progressive worsening of bilateral lower limb numbness and weakness, more pronounced on the left side; emergency thoracolumbar MRI showed no definite intraspinal hemorrhage, and decompressive edema was considered. On postoperative day 4, symptoms continued to deteriorate; repeat thoracolumbar MRI showed a left-sided clumped hematoma at the T10/11 level compressing the dural sac (Figures 2D,E). Emergency endoscopic intraspinal hematoma evacuation was performed on April 17, 2024. Intraoperatively, a dense hematoma was found within the original decompression range, compressing the dural sac and displacing it to the right. The hematoma was gradually stripped under endoscopic visualization, and the dural sac and nerve roots were confirmed to be relaxed and pulsating well.

Pathology: The first surgical pathology showed extensively calcified degenerative fibrocartilage tissue surrounded by dense fibrous connective tissue and hyaline degeneration. Lobulated tophaceous deposits were seen within the protruding nucleus pulposus tissue and adjacent epidural space, accompanied by multinucleated giant cells, foreign body giant cells, and chronic inflammatory cell infiltration. Polarized light microscopy showed lobulated tophaceous deposits with strongly birefringent urate crystals (Figures 2F,G). The second surgical pathology was dominated by fibrinoid and clot-like material, with massive acute and chronic inflammatory cell infiltration (predominantly neutrophils) and a small amount of keratinized and calcified tissue.

Postoperative management and follow-up: Postoperative rheumatology consultation confirmed the diagnosis of gout. Due to postoperative liver function fluctuations, febuxostat 20 mg/d was initiated after liver function improvement. At the 1-month follow-up (May 24, 2024), bilateral lower limb sensation and motor function had significantly recovered. Repeat thoracolumbar MRI showed postoperative changes at T10/11 (partial laminar defect), no obvious abnormal spinal cord signals, and no residual intraspinal lesions (Figure 2H).

### 4.2 Patient 2

A 21-year-old male was admitted to the spinal surgery department on May 14, 2024, with a 6-month history of waist and right lower limb soreness, numbness, and discomfort. He had a 1-year history of gout. Physical examination showed no obvious limitation of lumbar mobility, tenderness in the right iliolumbar muscles, numbness over the right lateral calf and little toe, slight flexion contracture of the right knee, and an antalgic gait. Laboratory tests showed serum uric acid of 836.0 μmol/L, ALT 243 U/L, and AST 113 U/L.

Imaging: Lumbar MRI showed a mass-like abnormal signal at the L5 level in the spinal canal, hypointense on T1WI and hypointense on T2WI, closely related to the cauda equina nerve roots, compressing the L5 and S1 nerve roots. The lesion was poorly demarcated from surrounding tissue, mainly located in the epidural space and involving the facet joints (Figures 1A,B). Contrast-enhanced MRI showed obvious eccentric enhancement at the upper margin of the lesion, with mild enhancement in the remaining portions and better delineation of boundaries than on non-contrast images (Figure 1C). Three-dimensional CT reconstruction of the lumbar spine showed a strip-like hyperdense lesion in the spinal canal at L4–S1, with a maximum axial cross-section of approximately 1.6 cm × 1.0 cm and a longitudinal diameter of approximately 5.0 cm; CT values were approximately 142–272 HU. Combined with the clinical context, tophaceous gout was highly suspected (Figures 1D–G). Preoperative right knee CT and MRI showed a hyperdense lesion in the joint space and a small amount of joint effusion, suggesting peripheral tophaceous gout deposition.

Surgery: On May 20, 2024, under general anesthesia, intraspinal lesion resection at L4–S1, L5 and S1 nerve root adhesion release, and L5–S1 pedicle screw implantation with vertebral fusion and internal fixation were performed. Under surgical microscope assistance, multiple white calcified deposits were seen in the facet joint spaces, and white, tough deposits were present in the epidural space. The nerve roots were encased and adherent to gouty granulomatous tissue. Intraoperative tissue was sent for rapid pathology, which reported gouty granuloma. Estimated intraoperative blood loss was approximately 300 mL, with no transfusion.

Pathology: Postoperative routine pathology showed pink amorphous/crystalline material deposition surrounded by multinucleated giant cells, foreign body giant cells, and histiocytic reactions, consistent with gouty foreign body granuloma (Figures 1N,O).

Postoperative management and follow-up: Postoperative rheumatology consultation confirmed gouty arthritis. Due to markedly abnormal liver function in the early postoperative period (ALT 243 U/L), febuxostat 20 mg/d was added before discharge after liver function improvement. Postoperative CT re-examination showed postoperative changes at L5, spinal canal enlargement, bone defect, and a small amount of gas in the intervertebral space after L5–S1 implantation (Figures 1H,I). Approximately 2 weeks postoperatively (early June 2024), the patient was readmitted due to sudden severe pain in both knees with low-grade fever. At this time, febuxostat had been initiated for approximately 1 week, and serum uric acid was 416 μmol/L; crystal-dissolution reaction-induced acute flare was considered. On June 14, 2024, left knee arthroscopic debridement was performed, revealing white, shiny crystals in the joint cavity, distributed in punctate and patchy patterns on the joint surface and cartilage. In September 2024, whole-body DECT assessment showed multiple small patchy tophaceous deposits around L5, the sacrum, and bilateral iliac wings (volume 27.67 cm^3^), as well as multiple small patchy deposits around both knees (volume 5.5 cm^3^) (Figures 1J,K). In October 2024, right knee arthroscopic debridement and synovectomy were performed due to right knee pain, revealing extensive tophaceous deposits in the synovium, patellofemoral cartilage, medial and lateral femoral condyles, and tibial plateau, with cartilage erosion and 2 free tophaceous bodies in the joint cavity. Pathology of the right knee synovium showed chronic inflammation with focal gouty granuloma.

The patient was prescribed febuxostat postoperatively but demonstrated poor compliance and had self-discontinued the medication; serum uric acid was 955.0 μmol/L at one follow-up visit. At the 20-month follow-up (January 2026), the patient presented with waist trauma; thoracic MRI showed compression fractures of T3, T5, T10, and T12 with bone marrow edema, T6/7/8/9/10 disc herniation, and possible thoracic spinal cord injury degeneration (Figure 1L). Lumbar CT showed postoperative changes in the lumbosacral spine and T12 compression fracture (Figure 1M). Given the concomitant history of lumbar trauma and the absence of preoperative bone mineral density data or dynamic DECT monitoring of bone erosion, these bone events should be interpreted as complications requiring long-term vigilance, in which long-term hyperuricemia may have exacerbated bone structural fragility against the background of trauma, rather than as proven causal outcomes of tophaceous gout.

The remaining three patients (Patients 3–5) shared similar clinical presentations of chronic back pain with lower limb neurological deficits and elevated serum uric acid levels, although they differed in affected spinal levels, preoperative diagnoses, and therapeutic strategies. Patient 3 was initially suspected of gouty spondylitis and underwent open decompression with 3-level fusion. Patient 4 was misdiagnosed as having lumbar disc herniation/stenosis and underwent open decompression and fusion. Patient 5 was suspected of malignancy/metastasis and received CT-guided biopsy only, followed by conservative management with urate-lowering therapy by rheumatology. Their comprehensive demographic, imaging, pathological, and follow-up data are provided in Table 1.

## 5 Systematic review of reported cases

The clinical characteristics of the 5 center cases are presented separately in Sections 3.1–3.4 of the Results and Section 4 (Case series) and in Table 1. In the pooled analysis below, these 5 cases were combined with the 33 literature cases (Supplementary Table 1). As of 2026, based on 30 studies (6–35) encompassing 33 literature cases and the 5 cases in this series, a total of 38 strictly defined intraspinal tophaceous gout cases were analyzed. Patient ages ranged from 16 to 73 years, with a mean of 45.1 years and a median of 47.5 years. Males accounted for the vast majority (32 cases, 84.2%), with a male-to-female ratio of approximately 5.3:1. Affected segment distribution (some patients had multilevel involvement): lumbar spine 23 cases (60.5%), thoracic spine 11 cases (28.9%), cervical spine 5 cases (13.2%). Anatomically, the lesions were located in the epidural space in 28 cases (73.7%), subdural/intramedullary in 4 (10.5%), unspecified intraspinal location in 4 (10.5%), and intradiscal in 2 (5.3%).

Due to the lack of specificity in imaging manifestations, the preoperative misdiagnosis rate of intraspinal tophaceous gout is extremely high. In the center cohort, 3 of the 5 patients (60%) were misdiagnosed preoperatively (Table 1). Among all 38 cases, 17 (44.7%) were initially considered to represent tumors or other malignant lesions, 9 (23.7%) as disc herniation/spinal stenosis, and 7 (18.4%) as infection/abscess, the remaining cases were either unspecified or suspected gout preoperatively. Regarding diagnosis, 36 cases (94.7%) were confirmed by postoperative histopathology, and 2 (5.3%) by needle biopsy without open surgery (one literature case managed conservatively and Patient 5 of this series).

## 6 Discussion

This study strictly defines intraspinal tophaceous gout as MSU crystal deposition located within the effective spinal canal volume (epidural, subdural, intramedullary, or intradiscal protrusion into the canal) presenting as a space-occupying lesion with neural compression as the primary manifestation, excluding lesions involving only the vertebral body/appendages/ligaments without intraspinal extension. The rationale for this strict definition is that intraspinal lesions differ fundamentally from vertebral/appendage lesions in terms of management strategy, misdiagnosis spectrum, and prognostic assessment criteria. All 5 cases in this cohort met the above strict definition and were characterized by a markedly younger age than previously reported cases. More importantly, despite all 5 patients having a documented history of gout, the preoperative misdiagnosis rate remained as high as 60%, indicating that clinicians still misdiagnose intraspinal involvement as disc herniation or tumor even in known gout patients.

It should be acknowledged that this strict definition has its limitations and applicability boundaries. Lesions confined to the ligamentum flavum without protrusion into the spinal canal were excluded from this study; however, isolated hypertrophy or calcification of the ligamentum flavum caused by gouty deposition can itself lead to spinal canal stenosis and neural compression (29, 30). Therefore, the conclusions of this study apply specifically to tophaceous deposits located within the effective spinal canal volume and should not be directly extrapolated to ligamentum flavum-dominant disease. A unified anatomical classification system for spinal gout that incorporates all axial compartments—including vertebral, paraspinal, ligamentum flavum, and truly intraspinal involvement—is needed in the future to standardize research inclusion criteria and guide clinical decision-making.

### 6.1 Diagnostic pitfalls and imaging strategy: From CT values to DECT stepwise assessment

The preoperative misdiagnosis rate of intraspinal tophaceous gout is extremely high, consistent with literature reports (approximately 45% of the pooled cases were initially misdiagnosed as tumor or malignancy). As illustrated by the contrasting imaging presentations of Cases 1 and 2 (see Section 3.2), the presence or absence of a measurable hyperdense component on CT directly determined whether gout could be suspected preoperatively, suggesting that routine CT value measurement is an important screening step for spinal canal-occupying lesions. This suggests that CT value measurement is an important preoperative screening tool: tophus CT values typically range between 150 and 400 HU, higher than most soft tissue tumors but lower than cortical bone, and can serve as a qualitative reference.

The high proportion of cases initially misdiagnosed as tumor or malignancy (approximately 50% of the pooled cases) deserves particular attention. Intraspinal tophaceous gout can present as a mass-like lesion with bone destruction and acute or progressive neurological deterioration, closely mimicking aggressive spinal neoplasms on initial imaging. Conversely, malignant spinal tumors may also produce rapid myelopathy or even paraplegia—most commonly through pathological vertebral fracture with epidural extension—making the distinction difficult on initial imaging alone. For example, acute paraplegia caused by a pathological fracture of the third thoracic vertebra due to metastatic melanoma has been reported to present with rapidly worsening back pain and progressive loss of motor and sensory function, a clinical picture hardly distinguishable from that of a destructive spinal mass lesion (36). These similarities underscore that, in patients presenting with a spinal canal-occupying lesion and neurological deficits, the diagnosis should be confirmed by histological examination—preferably by needle biopsy—whenever it remains uncertain, rather than proceeding directly on the assumption of malignancy. This consideration further supports the biopsy-first strategy discussed in Section 6.2 and the central role of pathological confirmation emphasized in this study.

However, CT values have significant limitations: they cannot distinguish urate deposits from other calcified or hyperdense lesions (e.g., osteophytes, calcified discs), cannot accurately assess the anatomical relationship between the lesion and neural structures, and cannot provide systemic urate burden information. In this context, DECT should serve as an upgraded assessment modality. Unlike MRI, which presents non-specific T1 hypointense and T2 hypo-/hyperintense signals that overlap extensively with schwannoma, meningioma, and inflammatory granuloma, DECT utilizes the unique photoelectric absorption characteristics of urate crystals to generate material decomposition color-coded maps, enabling non-invasive “histological-level” diagnosis preoperatively—a capability that conventional CT and MRI lack.

Furthermore, DECT has three irreplaceable functions in preoperative assessment: (1) Systemic disease burden evaluation: Intraspinal tophaceous gout is rarely an isolated event; DECT can map systemic urate deposition in a single scan, quantifying joint and tendon involvement to directly guide postoperative urate-lowering intensity. (2) Surgical roadmap function: DECT precisely delineates tophus calcification range, bone erosion boundaries, and anatomical relationships with the dural sac and nerve root exits, enabling surgeons to anticipate difficulty, select approach, and prepare for dural repair or spinal reconstruction. (3) Exclusionary diagnostic value: For atypical cases lacking typical peripheral gout history or with normal serum uric acid, a positive DECT result is decisive for establishing the diagnosis and can avoid invasive biopsy or exploratory surgery—particularly valuable for intramedullary/subdural lesions.

The extremely low preoperative DECT utilization rate in this series and the literature (7.9%, 3/38) does not reflect a lack of technical value, but rather insufficient clinical awareness of DECT's potential in spinal space-occupying lesions. Based on the theoretical analysis and the literature evidence presented in this study, we strongly suggest that DECT be considered for exploratory application in clinical practice in patients with MRI showing extramedullary intradural or epidural occupation with atypical signals, especially young-to-middle-aged men with hyperuricemia or gout history. A stepwise imaging strategy of “CT value screening, followed by DECT for precise diagnosis and systemic assessment” may therefore be explored. We acknowledge that none of the 5 center cases underwent preoperative DECT, and thus the preoperative value of DECT derived from our cohort remains a theoretical inference that requires prospective validation.

### 6.2 Individualized surgical strategy: neural compression dictates surgery, biopsy avoids overtreatment

Because intraspinal tophaceous gout occupies the effective spinal canal volume, it almost inevitably causes neurological dysfunction. Surgical decompression is the mainstay for relieving mechanical compression, as urate-lowering therapy alone cannot dissolve large established tophi in the short term. Consistent with our approach, a recent case report combined with bioinformatics analysis has also highlighted that a combined surgical and pharmacological strategy for gouty lumbar spinal stenosis yields a favorable prognosis in young patients (35). In this series, 4 cases (80%) underwent surgical decompression (3 open + 1 endoscopic), while only 1 patient underwent CT-guided needle biopsy due to strong preoperative suspicion of malignancy, with no further open surgery after confirmation.

An important finding of this study is that a biopsy-first strategy can prevent some patients from undergoing unnecessary tumor radical surgery. Case 5 was preoperatively suspected of malignancy/metastasis; direct open surgery would have entailed extensive laminectomy and possibly fusion fixation. Biopsy-confirmed gout avoided overtreatment. For spinal canal occupations with unclear preoperative diagnosis and strong suspicion of malignancy, a biopsy-first strategy is reasonable; however, once diagnosed, the decision for surgical decompression should still be based on the degree of neural compression. For cases with preoperative DECT-confirmed diagnosis, surgical planning can proceed directly without biopsy delay.

Surgical approach selection depends on the degree of neural compression and spinal stability: endoscopic/minimally invasive decompression is suitable for single-segment lesions without significant bony instability (Case 1), offering the advantages of minimal trauma and rapid recovery; open decompression with fusion and internal fixation is indicated for multilevel involvement with severe bone destruction (Cases 2, 3, 4), providing immediate stability but with correspondingly increased surgical trauma and blood loss. In this series, hematoma occurred after endoscopic decompression (Case 1), although its cause remains undetermined (see Section 3.4), this observation suggests that meticulous hemostasis and close postoperative neurological monitoring are warranted after endoscopic procedures, particularly in the presence of tophaceous tissue. Intraoperatively, excessive removal of normal bone in pursuit of complete tophus excision should be avoided to prevent iatrogenic spinal instability. Postoperative pathological confirmation remains the gold standard; intraoperative frozen section pathology (if available) can assist in guiding surgical extent.

### 6.3 Postoperative management and long-term prognosis: first report of hematoma and bone metabolism monitoring

This series reports a postoperative intraspinal hematoma (Case 1), a rare complication not previously described in the literature. Because the first operation was endoscopic decompression and discectomy, and no vascular pathological evidence was obtained, the origin of this hematoma cannot be definitively determined: it may have resulted from delayed bleeding related to gouty erosion of blood vessels (e.g., gouty vasculitis), but a technical complication of the surgical procedure itself (e.g., incomplete endoscopic hemostasis) or a local coagulation abnormality cannot be excluded. Once diagnosed, emergency hematoma evacuation with neural decompression is indicated. For patients undergoing endoscopic surgery, close vigilance for delayed neurological deterioration within the first postoperative week (particularly POD 1–5) is essential, with MRI re-examination if necessary.

Long-term compliance with urate-lowering therapy directly affects prognosis. In Case 2, poor compliance with febuxostat was followed by multiple vertebral compression fractures and thoracic spinal cord injury at 20 months postoperatively, which should be regarded as a complication requiring long-term vigilance rather than a proven causal outcome of hyperuricemia, particularly given the background of trauma. Although not routine preoperatively, DECT can serve as a postoperative tool for quantifying systemic tophus burden and monitoring the efficacy of urate-lowering therapy. Additionally, for young patients, potential genetic predisposition to early-onset severe gout should be considered, and genetic counseling is recommended when appropriate. Finally, it should be emphasized that gout is a systemic metabolic disease, and neural compression caused by tophaceous deposition is not limited to the spine. Peripheral nerve entrapment—most commonly carpal tunnel syndrome resulting from tophaceous infiltration of the flexor tendons or even the epineurium of the median nerve—has also been increasingly reported, occasionally as the first clinical manifestation of gout (37). This observation provides an additional rationale for the systemic metabolic control emphasized in this study and for the use of DECT to quantify the overall urate burden rather than focusing solely on the spinal lesion.

### 6.4 Clinical practice implications

Based on this study, we propose the following recommendations for orthopedic and rheumatology colleagues: (1) Preoperative vigilance: For young-to-middle-aged men presenting with radiculopathy or myelopathy, intraspinal tophaceous gout should be included in the differential diagnosis regardless of prior gout history. (2) Stepwise imaging evaluation: For atypical spinal canal occupations on MRI, initial CT value analysis should be performed; for suspected cases, DECT may be applied in an exploratory manner to establish the diagnosis, assess the systemic burden, and guide surgical planning. Given that preoperative DECT was not performed in our own cohort, its preoperative value warrants further prospective validation. (3) Metabolic-surgical integrated management: Urate-lowering therapy should be initiated within 1–2 weeks postoperatively (target serum uric acid <300 μmol/L), with rheumatology responsible for long-term follow-up and DECT systemic monitoring, and spinal surgery responsible for operative site imaging follow-up. Establishing a multidisciplinary collaborative pathway is essential to reduce recurrence risk and improve long-term prognosis.

## 7 Limitations

This study has several limitations. First, as a retrospective case series from two centers with a small sample size, selection bias is inevitable. Second, the literature review was limited to English-language databases (PubMed and Embase), and non-English cases may have been missed. Third, the lack of preoperative DECT in all 5 cases precluded direct validation of the proposed stepwise imaging strategy within our own cohort. Fourth, the follow-up duration was variable, and long-term outcomes for all patients remain incomplete. Fifth, preoperative urate-lowering status and the time to postoperative urate target attainment were not systematically documented, which precluded a formal analysis of the relationship between urate control and outcomes. Finally, the pathophysiological mechanism of postoperative intraspinal hematoma in gout patients requires further investigation.

## 8 Conclusion

Intraspinal tophaceous gout is a rare subtype of spinal gout. Under a strict anatomical definition, this study presents a 5-case series characterized by a notably younger patient age than previously reported. The main conclusions are as follows: (1) Even with a documented history of gout, the preoperative misdiagnosis rate of intraspinal tophaceous gout remains high; CT value analysis is an important tool for reducing misdiagnosis. (2) Surgical decompression is the mainstay for relieving neural compression, while biopsy-first strategies can prevent unnecessary tumor radical surgery in some patients. (3) Postoperative intraspinal hematoma is a rare but critical complication requiring vigilance. (4) Long-term urate-lowering therapy and bone metabolism monitoring are essential for improving prognosis. Multidisciplinary collaboration is the recommended management model for such patients.

## Data Availability

The raw data are available from the corresponding author upon reasonable request.
